# Supplementary figures and images for: The efficiency of rotavirus A spread to extraintestinal tissues is not determined by the levels of its replication in the gut
Source: PLoS Pathog. 2025 Nov 25;21(11):e1013723. doi: 10.1371/journal.ppat.1013723 (PMC12674514; doi:10.1371/journal.ppat.1013723)

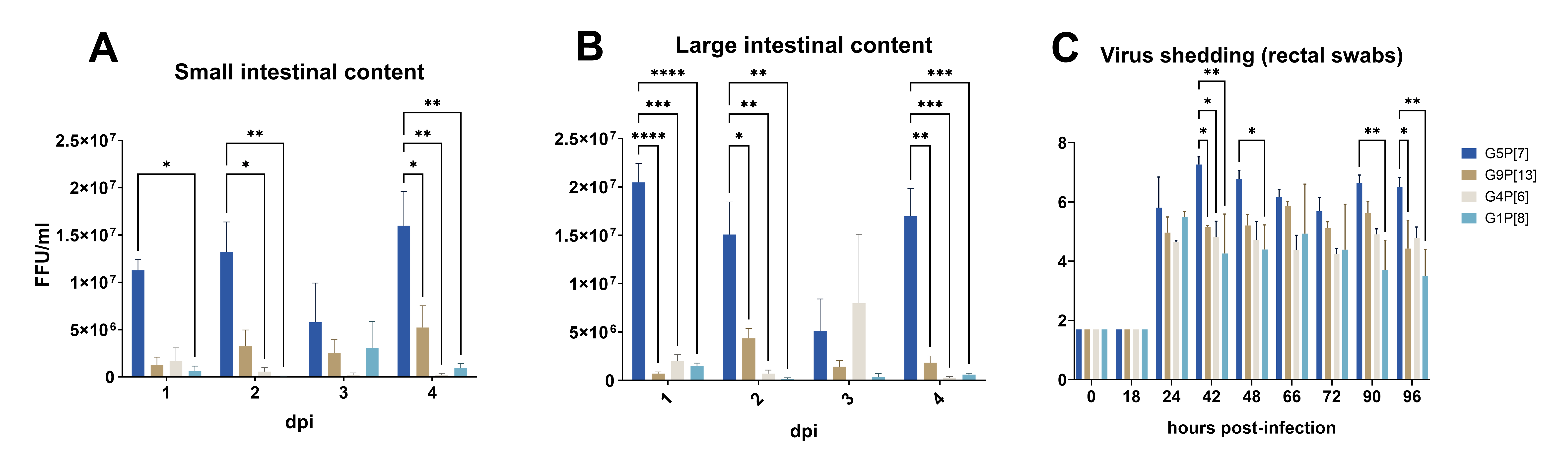

Supplement: S1 Fig — Six-day-old germ-free pigs were orally inoculated with 1 × 10^6^ FFU of rotavirus. Swabs were collected at designated time points. For the small and large intestine contents pigs were euthanized at the post-inoculation times indicated and contents were collected. RVA quantification was performed with cell culture immunofluorescence (CCIF). Significant differences (*p < 0.05, ** p < 0.01, p < 0.001) are indicated as calculated by using two-way ANOVA with repeated measures and the Tukey-Kramer test for multiple comparisons. (TIF) [file ppat.1013723.s001.tif]

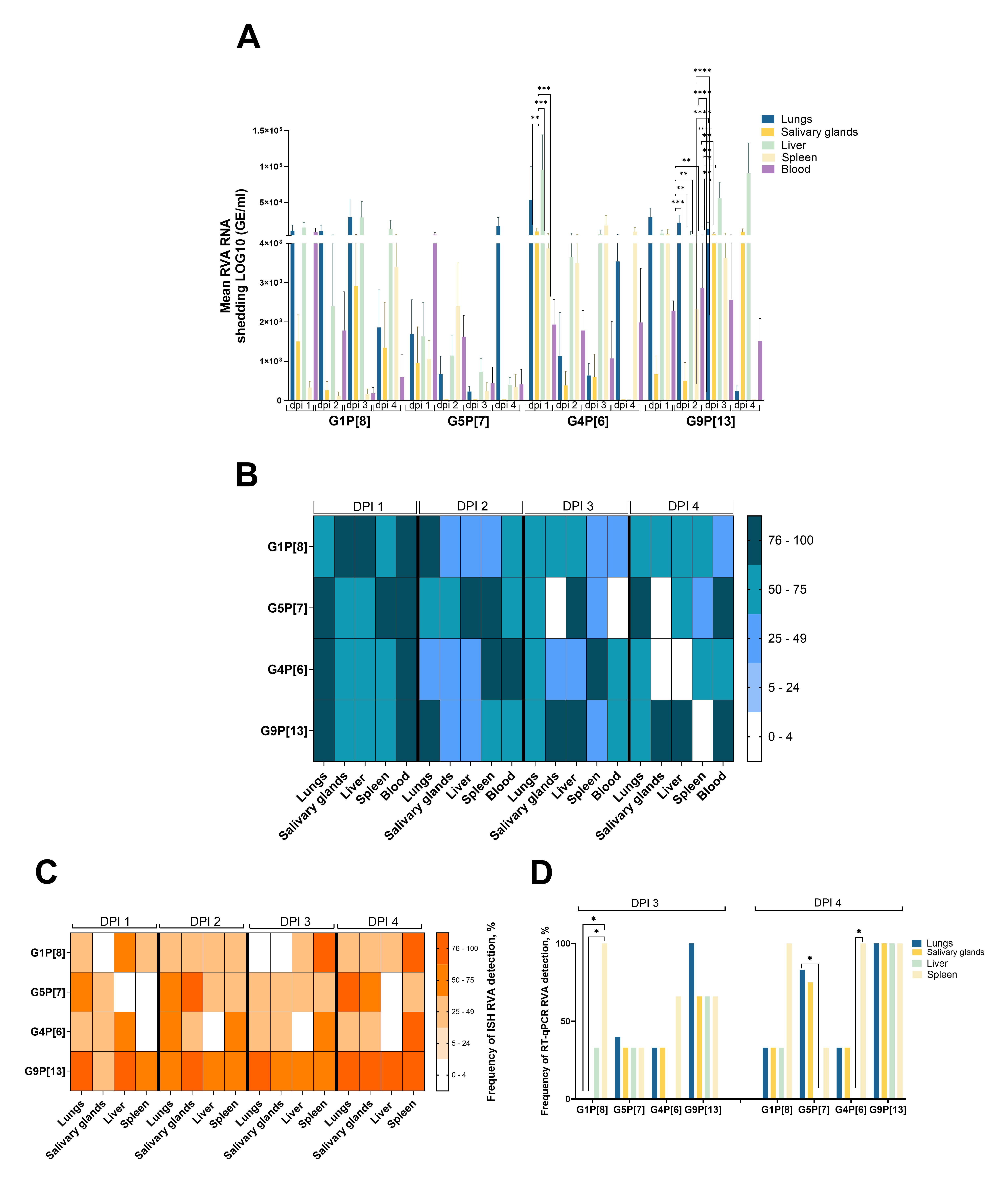

Supplement: S2 Fig — Six-day-old germ-free pigs were orally inoculated with 1 × 10^6^ FFU of rotavirus. At the post inoculation times indicated, animals were euthanized and tissues (lungs, salivary glands, liver, blood and spleen) were collected. (A) Levels of RVA RNA in extraintestinal tissues across dpi 1–4. (B) RT-qPCR RVA RNA detection frequency in extraintestinal tissues across dpi 1–4. (C) ISH RT-qPCR RVA RNA detection frequency in extraintestinal tissues across dpi 1–4 and (D) at time points when significant differences were observed. Statistical analysis for RVA RNA detection frequency was performed by using Fisher’s exact test. Significant differences (*p < 0.05) are indicated. (TIF) [file ppat.1013723.s002.tif]

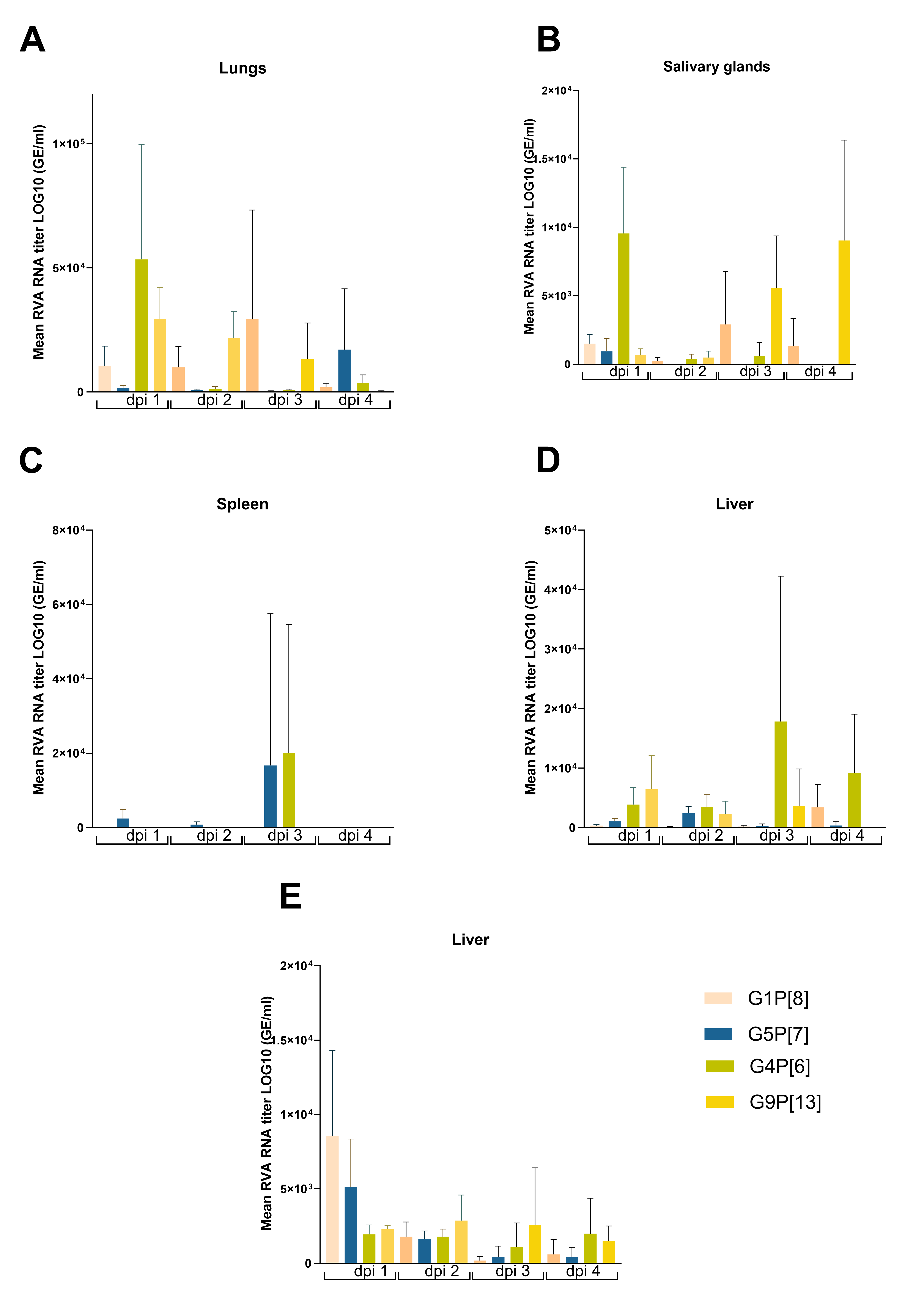

Supplement: S3 Fig — Six-day-old germ-free pigs were orally inoculated with 1 × 10⁶ FFU of each rotavirus. At the indicated post-inoculation time points, animals were euthanized, and tissues (A: lungs; B: salivary glands; C: liver; D: spleen and E: blood) were collected. Significant differences (*p < 0.05, **p < 0.01) were determined using two-way ANOVA with repeated measures and the Tukey–Kramer test for multiple comparisons. (TIF) [file ppat.1013723.s003.tif]

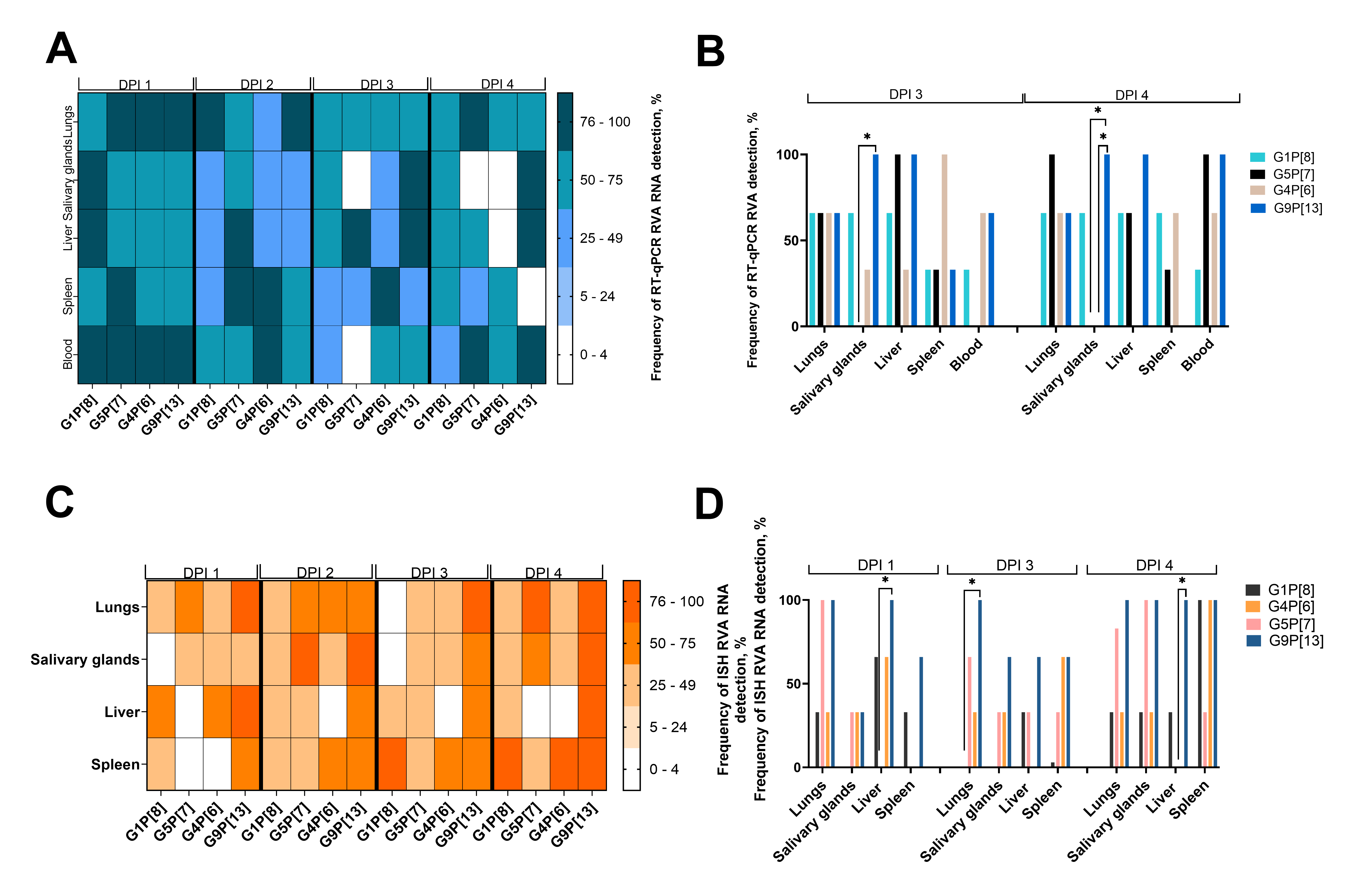

Supplement: S4 Fig — Six-day-old germ-free pigs were orally inoculated with 1 × 10⁶ FFU of each rotavirus. At the indicated post-inoculation time points, animals were euthanized, and tissues (lungs, salivary glands, liver, blood, and spleen) were collected. (A) RT-qPCR RVA RNA detection frequency across dpi 1–4; (B) Detection frequency at time points where significant differences were observed. The frequency of RVA detection across tissues from dpi 1–4 was assessed using Fisher’s exact test. Significant differences (*p < 0.05) are indicated. (C) ISH-based RVA RNA detection frequency across dpi 1–4; (D) Detection frequency at time points with significant differences. Statistical analysis was performed using Fisher’s exact test. Significant differences (*p < 0.05) are indicated. (TIF) [file ppat.1013723.s004.tif]

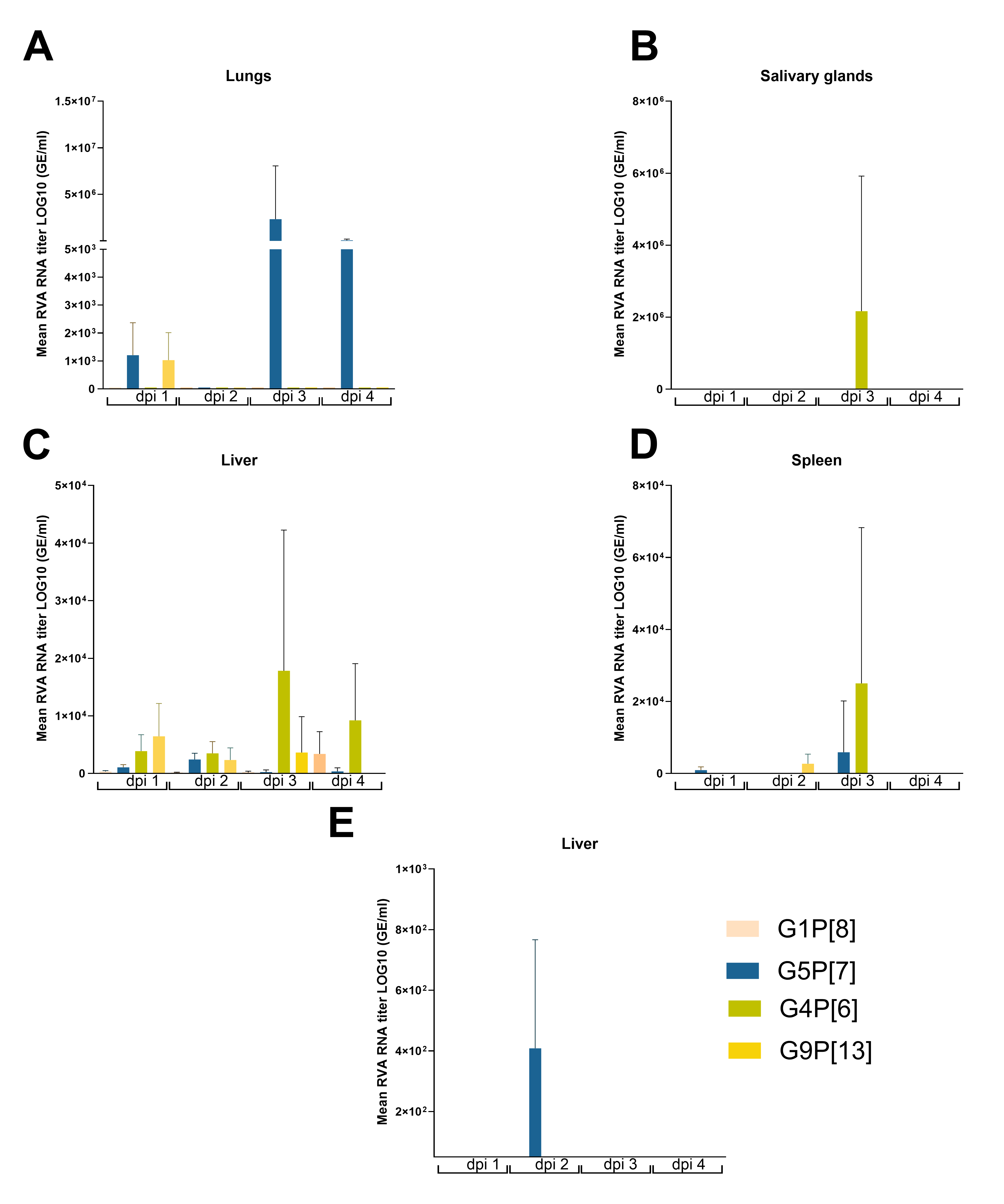

Supplement: S5 Fig — Six-day-old germ-free pigs were orally inoculated with 1 × 10⁶ FFU of each rotavirus. At the indicated post-inoculation time points, animals were euthanized, and tissues (A: lungs; B: salivary glands; C: liver; D: spleen and E: blood) were collected. Significant differences (*p < 0.05, **p < 0.01) were determined using two-way ANOVA with repeated measures and the Tukey–Kramer test for multiple comparisons. (TIF) [file ppat.1013723.s005.tif]

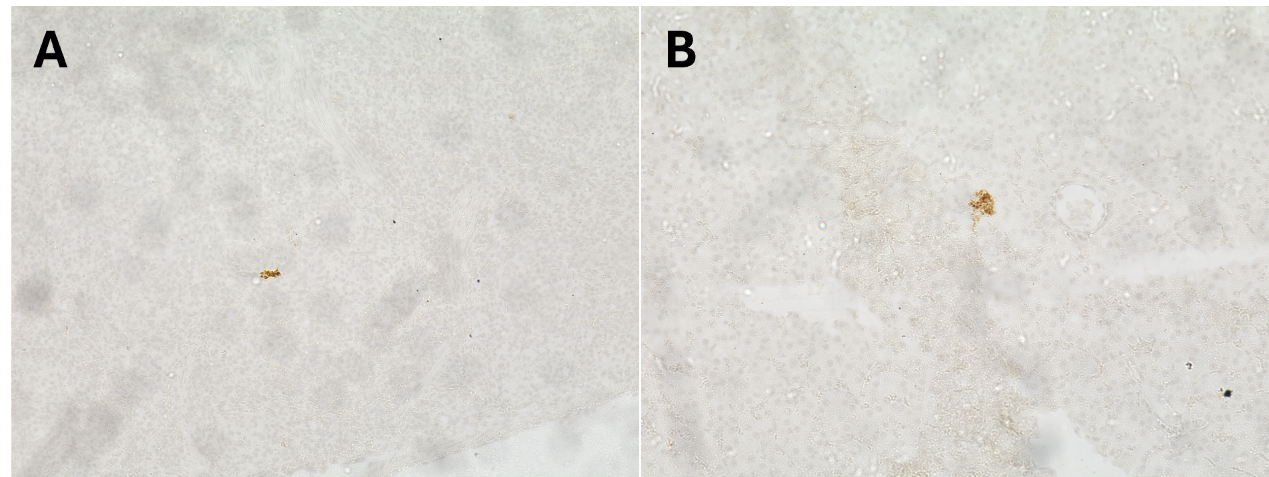

Supplement: S1 Picture — (TIF) [file ppat.1013723.s008.tif]
