# Supplementary material for: The efficiency of rotavirus A spread to extraintestinal tissues is not determined by the levels of its replication in the gut
Source: PLoS Pathog. 2025 Nov 25;21(11):e1013723. doi: 10.1371/journal.ppat.1013723 (PMC12674514; doi:10.1371/journal.ppat.1013723)
Supplement: S1 Table — (DOCX) [file ppat.1013723.s006.docx]

| **Rotavirus A strain** | **Days post infection** | | | | **Total** |
| --- | --- | --- | --- | --- | --- |
|  | **1** | **2** | **3** | **4** |  |
| Wa G1P[8] | 3 | 3 | 3 | 3 | 12 |
| RV0084 G9P[13] | 3 | 3 | 3 | 3 | 12 |
| Gottfried G4P[6] | 3 | 3 | 3 | 3 | 12 |
| OSU G5P[7] | 6 | 6 | 6 | 6 | 24 |
| control | 1 | 1 | 1 | 1 | 4 |
