# Supplementary material for: The efficiency of rotavirus A spread to extraintestinal tissues is not determined by the levels of its replication in the gut
Source: PLoS Pathog. 2025 Nov 25;21(11):e1013723. doi: 10.1371/journal.ppat.1013723 (PMC12674514; doi:10.1371/journal.ppat.1013723)
Supplement: S2 Table — (DOCX) [file ppat.1013723.s007.docx]

| Target | Primer/probe | Sequence, 5′-3′ | Reference |
| --- | --- | --- | --- |
| NSP3 | Forward | ACCATCTACACATGACCCTC | Pang XL, Lee B, Boroumand N, Leblanc B, Preiksaitis JK, Yu Ip CC. Increased detection of rotavirus using a real time reverse transcription-polymerase chain reaction (RT-PCR) assay in stool specimens from children with diarrhea. J Med Virol. 2004 Mar;72(3):496-501. doi: 10.1002/jmv.20009. PMID: 14748075. |
|  | Reverse | GGTCACATAACGCCCC |  |
|  | Probe | FAM-ATGAGCACAATAGTTAAAAGCTAACACTGTCAA-TAM |  |
