# Supplementary material for: The efficiency of rotavirus A spread to extraintestinal tissues is not determined by the levels of its replication in the gut
Source: PLoS Pathog. 2025 Nov 25;21(11):e1013723. doi: 10.1371/journal.ppat.1013723 (PMC12674514; doi:10.1371/journal.ppat.1013723)
Supplement: Attachment — Submitted filename: Response to reviewers.docx [file ppat.1013723.s010.docx]

## Editor’s comments.

1. Please ensure that the CRediT author contributions listed for every co-author are completed accurately and in full. At this stage, the following Authors/Authors require contributions: Sergei A Raev, Maryssa K Kick, Maria Chellis, Linda J Saif, Talita P Resende, and Anastasia Nickolaevna Vlasova. Please ensure that the full contributions of each author are acknowledged in the "Add/Edit/Remove Authors" section of our submission form.
   The list of CRediT author contributions may be found here: [https://journals.plos.org/plospathogens/s/authorship#loc-author-contributions](https://urldefense.com/v3/__https:/journals.plos.org/plospathogens/s/authorship*loc-author-contributions__;Iw!!KGKeukY!x4wQK-2H9kiZ_9l0s89J3DF70McG9m0lXtH6kdX_fZTre1t-emzreIVUBwx4VW0iyy7CjlrgTyalQu95nQ$)

AU: The authors declare that there are no competing interests.

2. We ask that a manuscript source file is provided at Revision. Please upload your manuscript file as a .doc, .docx, .rtf or .tex. If you are providing a .tex file, please upload it under the item type u2018LaTeX Source Fileu2019 and leave your .pdf version as the item type u2018Manuscriptu2019.

AU:

3. We do not publish any copyright or trademark symbols that usually accompany proprietary names, eg ©,  ®, or TM  (e.g. next to drug or reagent names). Therefore please remove all instances of trademark/copyright symbols throughout the text, including:
   - TM on page: 15.

AU: Revised as suggested

4. Please upload all main figures as separate Figure files in .tif or .eps format. For more information about how to convert and format your figure files please see our guidelines:
   [https://journals.plos.org/plospathogens/s/figures](https://urldefense.com/v3/__https:/journals.plos.org/plospathogens/s/figures__;!!KGKeukY!x4wQK-2H9kiZ_9l0s89J3DF70McG9m0lXtH6kdX_fZTre1t-emzreIVUBwx4VW0iyy7CjlrgTya76CR8YA$)

AU: Revised as suggested

5. We notice that your supplementary Figures, and Tables are included in the manuscript file. Please remove them and upload them with the file type 'Supporting Information'. Please ensure that each Supporting Information file has a legend listed in the manuscript after the references list.

AU: Revised as suggested

6. Some material included in your submission may be copyrighted. According to PLOSu2019s copyright policy, authors who use figures or other material (e.g., graphics, clipart, maps) from another author or copyright holder must demonstrate or obtain permission to publish this material under the Creative Commons Attribution 4.0 International (CC BY 4.0) License used by PLOS journals. Please closely review the details of PLOSu2019s copyright requirements here: PLOS Licenses and Copyright. If you need to request permissions from a copyright holder, you may use PLOS's Copyright Content Permission form. Please respond **directly to this email** and provide any known details concerning your material's license terms and permissions required for reuse, even if you have not yet obtained copyright permissions or are unsure of your material's copyright compatibility. Once you have responded and addressed all other outstanding technical requirements, you may resubmit your manuscript within Editorial Manager. Potential Copyright Issues:i) Please confirm (a) that you are the photographer of 5, or (b) provide written permission from the photographer to publish the photo(s) under our CC BY 4.0 license. ii) Please confirm (a) that you are the photographer of 5, or (b) provide written permission from the photographer to publish the photo(s) under our CC BY 4.0 license.

AU: I confirm that I am the photographer of all five photos, and I grant permission to publish them under the CC BY 4.0 license.

7. We note that figure 7 is created through BioRender. Please confirm that you hold a Premium account and provide a pdf copy of the CC BY 4.0 Licence as provided by BioRender. For instructions on how to generate a CC BY 4.0 license for your figure, please see the guidelines here: [https://help.biorender.com/hc/en-gb/articles/21282341238045-Publishing-in-open-access-resources](https://urldefense.com/v3/__https:/help.biorender.com/hc/en-gb/articles/21282341238045-Publishing-in-open-access-resources__;!!KGKeukY!x4wQK-2H9kiZ_9l0s89J3DF70McG9m0lXtH6kdX_fZTre1t-emzreIVUBwx4VW0iyy7CjlrgTya5Z0L1Ig$). If you are using the free assets from BioRender, we are unable to publish these images as they are licenced under a stricter licence than CC BY 4.0. In this case we ask you to remove the BioRender images and replace them with open source alternatives. See these open source resources you may use to replace images / clip-art:
   - [https://bioart.niaid.nih.gov/](https://urldefense.com/v3/__https:/bioart.niaid.nih.gov/__;!!KGKeukY!x4wQK-2H9kiZ_9l0s89J3DF70McG9m0lXtH6kdX_fZTre1t-emzreIVUBwx4VW0iyy7CjlrgTybQZkpzWQ$) 
   - [https://bioicons.com/](https://urldefense.com/v3/__https:/bioicons.com/__;!!KGKeukY!x4wQK-2H9kiZ_9l0s89J3DF70McG9m0lXtH6kdX_fZTre1t-emzreIVUBwx4VW0iyy7CjlrgTyZ73Bi-uA$)
   - [https://healthicons.org/](https://urldefense.com/v3/__https:/healthicons.org/__;!!KGKeukY!x4wQK-2H9kiZ_9l0s89J3DF70McG9m0lXtH6kdX_fZTre1t-emzreIVUBwx4VW0iyy7CjlrgTyYB8nImYQ$) 
   - [https://scidraw.io/](https://urldefense.com/v3/__https:/scidraw.io/__;!!KGKeukY!x4wQK-2H9kiZ_9l0s89J3DF70McG9m0lXtH6kdX_fZTre1t-emzreIVUBwx4VW0iyy7CjlrgTyaY_R_cdg$)
   - [https://reactome.org/icon-lib](https://urldefense.com/v3/__https:/reactome.org/icon-lib__;!!KGKeukY!x4wQK-2H9kiZ_9l0s89J3DF70McG9m0lXtH6kdX_fZTre1t-emzreIVUBwx4VW0iyy7CjlrgTyb4l4_n7A$)
   - [https://www.phylopic.org/images](https://urldefense.com/v3/__https:/www.phylopic.org/images__;!!KGKeukY!x4wQK-2H9kiZ_9l0s89J3DF70McG9m0lXtH6kdX_fZTre1t-emzreIVUBwx4VW0iyy7CjlrgTyaNEuxvfQ$) 
   - [https://journals.plos.org/plosbiology/article?id=10.1371/journal.pbio.3002395](https://urldefense.com/v3/__https:/journals.plos.org/plosbiology/article?id=10.1371*journal.pbio.3002395__;Lw!!KGKeukY!x4wQK-2H9kiZ_9l0s89J3DF70McG9m0lXtH6kdX_fZTre1t-emzreIVUBwx4VW0iyy7CjlrgTyaGBzFm0Q$)

AU: I confirm that we have a Premium account and a pdf copy of the CC BY 4.0 provided by Biorender is attached.

8. We note that your Data Availability Statement is currently as follows: "All relevant data are within the manuscript.". Please confirm at this time whether or not your submission contains all raw data required to replicate the results of your study. Authors must share the “minimal data set” for their submission. PLOS defines the minimal data set to consist of the data required to replicate all study findings reported in the article, as well as related metadata and methods ([https://journals.plos.org/plosone/s/data-availability#loc-minimal-data-set-definition](https://urldefense.com/v3/__https:/journals.plos.org/plosone/s/data-availability*loc-minimal-data-set-definition__;Iw!!KGKeukY!x4wQK-2H9kiZ_9l0s89J3DF70McG9m0lXtH6kdX_fZTre1t-emzreIVUBwx4VW0iyy7CjlrgTyYpUjBFmg$)).
   For example, authors should submit the following data: 
   1) The values behind the means, standard deviations and other measures reported;
   2) The values used to build graphs;
   3) The points extracted from images for analysis..
   Authors do not need to submit their entire data set if only a portion of the data was used in the reported study.
   If your submission does not contain these data, please either upload them as Supporting Information files or deposit them to a stable, public repository and provide us with the relevant URLs, DOIs, or accession numbers. For a list of recommended repositories, please see [https://journals.plos.org/plosone/s/recommended-repositories](https://urldefense.com/v3/__https:/journals.plos.org/plosone/s/recommended-repositories__;!!KGKeukY!x4wQK-2H9kiZ_9l0s89J3DF70McG9m0lXtH6kdX_fZTre1t-emzreIVUBwx4VW0iyy7CjlrgTya0d_f9Kw$). 
   If there are ethical or legal restrictions on sharing a de-identified data set, please explain them in detail (e.g., data contain potentially sensitive information, data are owned by a third-party organization, etc.) and who has imposed them (e.g., an ethics committee). Please also provide contact information for a data access committee, ethics committee, or other institutional body to which data requests may be sent. If data are owned by a third party, please indicate how others may request data access.

AU: Thank you for your comment. We confirm that all data required to replicate the results of our study are available. The Excel file containing the raw data (including values underlying all means, standard deviations, and other measures reported in the manuscript, as well as data used to construct graphs and figures) has now been uploaded as Supporting Information. This dataset includes all values used for statistical analyses and figure generation. No additional proprietary or ethically restricted data were used in this study.

9. Please amend your detailed Financial Disclosure statement. This is published with the article. It must therefore be completed in full sentences and contain the exact wording you wish to be published.
   1) State the initials, alongside each funding source, of each author to receive each grant. For example: "This work was supported by the National Institutes of Health (####### to AM; ###### to CJ) and the National Science Foundation (###### to AM)."
   2) State what role the funders took in the study. If the funders had no role in your study, please state: "The funders had no role in study design, data collection and analysis, decision to publish, or preparation of the manuscript."
   3) If any authors received a salary from any of your funders, please state which authors and which funders..
   If you did not receive any funding for this study, please simply state: u201cThe authors received no specific funding for this work.u201d

AU: Revised as suggested

10. Kindly revise your competing statement to align with the journal's style guidelines: 'The authors declare that there are no competing interests.'

AU: Revised as suggested

## Part I - Summary

1) Reviewer #1: This paper shows that RVA's extraintestinal spread does not depend on its replication efficiency in the gut, which may explain the current global dominance of G9P[13] and related RVA strains. In particular, the authors show that the G9P[3] strain could be capable of airborne spread, as the lungs have the highest frequency of RVA RNA detection, the highest host glycan diversity/abundance, and the presence of lesions both macroscopically and histologically. The authors' claims are substantiated by their well-structured experiments and research results, making this paper worthy of publication in PLoS Pathogens.

AU: We sincerely thank the reviewer for their thorough evaluation and positive comments on our work. We are pleased that the experiments and results were found to be well-structured and that the significance of our findings was recognized.

2) Reviewer #2: This manuscript investigates the extraintestinal dissemination of Rotavirus A (RVA) in gnotobiotic pigs, focusing on correlation between viral replication in the gut and its spread to other organs. The authors utilized four RVA strains with differing replication capacities—human Wa (G1P[8]), porcine RV0084 (G9P[13]), Gottfried (G4P[6]), and OSU (G5P[7]). Remarkably, the G9P[13] strain, which replicated to a lesser extent in the intestine, exhibited more efficient dissemination to extraintestinal tissues. These findings suggest that RVA extraintestinal spread is not determined by intestinal replication efficiency and that viral genomes detected in peripheral organs are not merely residual byproducts of intestinal replication. Furthermore, the authors demonstrate clear macroscopic and microscopic lesions in the lungs, suggesting that RVA infection may contribute to respiratory pathology—consistent with earlier observations in rodent models. However, several limitations in the dataset make it difficult to draw definitive conclusions regarding true extraintestinal replication. In particular, the heatmap-style data presentation obscures the time-course dynamics of viral genome levels in each organ—information that is critical to distinguishing between residual viral RNA and active replication. Additionally, no data on the presence of infectious virus (e.g., focus-forming units) in extraintestinal tissues are provided, making it difficult to assess whether productive infection occurred. While extraintestinal dissemination of RVA has been reported in neonatal mouse, rat, and piglet models (as cited in the manuscript), this study is unique in demonstrating consistent pathological changes in the lungs of infected piglets. Nonetheless, further evidence is needed to confirm that these changes are due to active viral replication and to clarify the functional significance of the observed pathology.

AU: We thank the reviewer for their detailed and insightful comments. We agree that demonstrating active extraintestinal replication is critical. To address this, we used in situ hybridization (ISH) in the lungs and other extraintestinal tissues, which provides direct evidence of active viral replication rather than residual viral RNA. It is possible because the probes used in this assay are designed to detect mRNA and not simply genomic RNA ([doi.org/10.1177/1040638718817502](https://doi.org/10.1177/1040638718817502)). We also acknowledge the concern regarding the heatmap presentation. In the revised manuscript, we have replaced the heatmaps with standard graphs depicting the time-course dynamics of viral genome levels in each organ. This will more clearly illustrate the progression of viral replication over time and help distinguish between residual RNA and active infection. Finally, to further confirm both the presence of actively replicating virus in extraintestinal tissues and its sporadic detection, consistent with earlier reports (Crawford et al., 2006), we have added infectious virus quantification from extraintestinal tissues to the supplementary data (Supplementary Figure 5).

3) Reviewer #3: In the current study, Raev et al. orally inoculated gnotobiotic piglets with three porcine (G5P[7], G9P[13], and G4P[6]) and one human (G1P[8]) species A rotavirus (RVA). They hypothesized that RVAs that replicate more efficiently in the gut should spread more efficiently to extraintestinal sites. They characterized viral replication, the presence of viral RNA, and signs of pathology in the intestine and at sites of dissemination, with a focus on two of the viruses (G5 and G9). Rectal virus shedding was higher for G5 than G9 (significant at some time points). However, RTqPCR and RNAish suggested a trend towards more frequent and/or higher levels of extraintestinal dissemination, particularly to the lungs, liver and salivary glands, for G9 than G5. The authors investigated the presence of several glycans known to serve as receptors for some RVAs in various piglet tissues and found the highest levels and diversity of these molecules present in the ileum followed by the lung. Evidence of gross pathology was detected in the lung, histological changes and viral antigen were detected in the lung and gut, and viral antigen was detected in the liver for at least some of the viruses, although the frequencies for each virus are unclear. Limited numbers of labs possess the capacity to conduct rotavirus infection studies in gnotobiotic piglets. The study found, unexpectedly, that the efficiency of RVA intestinal replication does not necessarily correlate with the efficiency of extraintestinal spread. This finding is significant and interesting and seems to be generally supported by the results. However, by more transparently displaying data points and subsets, the authors might improve confidence in this conclusion. Detection of higher levels of several sialylated and non-sialylated glycans that are receptors for some RVAs in piglet ileum and lungs is consistent with the reasonable and testable hypothesis that virus attachment is an important driver of the observed spread and pathology outcomes. However, this is a biased approach, and other viral and host factors, such as increased presence or diversity of proteases in the gut and lungs or differing sensitivities of viral activation by such proteases, could also explain the outcomes. Since the authors do not directly test the roles of specific glycans in virus attachment, at minimum, alternative explanations should be discussed. As definitive findings from the study are limited, it is essential that the methods are rigorous, and data are presented clearly. However, in several cases, the methods and figure captions lack details that are necessary or would be helpful for interpreting the findings.

AU: We sincerely thank the reviewer for their thoughtful evaluation and constructive suggestions. We are pleased that the novelty and significance of our findings were recognized. We agree that more transparent data presentation will improve confidence in our conclusions. In the revised manuscript, we have revised the figures to display individual data points and subsets more clearly, thereby allowing readers to better assess variability and trends across experimental groups. Regarding glycans, we concur that while our findings support a role for sialylated and non-sialylated glycans in viral attachment and dissemination, other viral and host factors (e.g., protease activity and activation mechanisms) may also contribute to the observed outcomes. We have incorporated this broader perspective into the Discussion to acknowledge these alternative explanations and highlight the need for further mechanistic studies. Finally, we acknowledge the importance of methodological transparency. We have expanded the Materials and Methods section and revise figure captions to include additional details necessary for interpretation, including sample sizes, experimental replicates, and specific conditions used.

*In addition to the presence of RVA receptors, other host factors—such as trypsin-like serine proteases(33)—may contribute to viral replication and tissue tropism. These proteases, abundant in the porcine intestine and required for RVA VP4 cleavage, are also present at lower levels in tissues like the lung, salivary glands, and liver(34,35). Other classes of proteases, including threonine hydrolases(36), have also been implicated in viral protein processing in certain tissues, though their specific role in RVA activation remains to be explored. Thus, limited extraintestinal replication may result not only from receptor availability but also from the local abundance and diversity of activating proteases, highlighting additional mechanisms that could explain the observed viral spread.*

Part II – Major Issues: Key Experiments Required for Acceptance

1) Reviewer #1: No further key experiments are required for acceptance.

AU: We thank the reviewer for their positive evaluation and for confirming that no additional experiments are required for acceptance.

2) Reviewer #2: The current data are insufficient to conclusively demonstrate that RVA replicates in extraintestinal organs. As the authors note in the discussion, viral genome copy numbers in these tissues were much lower than in the intestine. It remains unclear whether these represent true replication or are simply residual from systemic dissemination. Please consider addressing the following:
   - Provide time-course plots of viral genome copy numbers for each organ and virus strain individually (redrawing of Figures 1A and 3A) to assess whether viral RNA increases independently of intestinal replication. Avoid using heatmaps for primary quantitative data.
   - Include data on the detection of infectious virus (e.g., focus-forming units) in extraintestinal tissues, particularly the lungs.

AU: We appreciate the reviewer’s suggestion. As heatmaps for viral genome copy numbers were used in Figures 2A and 3A, we have redrawn these figures as individual time-course plots for each organ and virus strain. These plots now allow direct assessment of whether viral RNA increases in extraintestinal tissues independently of intestinal replication. Additionally, we have included the focus-forming data in Supplementary Figure 5 to provide complementary evidence of viral replication.

3) Reviewer #3: Figs 2,3, S2, and S3 report viral RNA levels in genome equivalents quantified by RT-qPCR, but details of the method are not reported and could not be found in the cited reference. Were identical primers used to quantify all rotavirus strains? These primers were designed and initially validated more then 20 years ago (reference has been updated) and have been in use by multiple research groups including ours. Was there a control for specificity, such as RNA from uninfected pigs or PIEs? How was the standard curve generated to quantify genome equivalents? If this information cannot be provided, the authors might need to conduct limited experiments to validate the method.

AU: We thank the reviewer for this suggestion. We have now added the following details into the Materials and Methods to clarify the RT-qPCR procedure:

*The 87 bp RVA-specific NSP3 sequence (Supplementary Table 2) was amplified and cloned using the TOPO TA kit (Thermo Fisher Scientific, MA, USA). Linearized plasmid was quantified by NanoDrop® 2000 and serially diluted (1 × 10¹⁰ to 0.25 copies/µL) with three replicates per dilution to generate a standard curve. Identical primers were used for all RVA strains. RNA from RVB, RVC, and uninfected piglets or PIEs served as negative controls. The standard curve had a slope of –3.053 (PCR efficiency 113%) and R² = 0.998, confirming highly linear amplification.*

In the ‘Minor Issues’ section, several suggestions are made regarding data presentation. These include the addition of individual data points to several graphs and information and panels to gross pathological and histological data figures. It is likely that the authors possess the necessary data to make these modifications and will not need to conduct additional experiments to address the comments. If the requested information is provided and rationalized, it is likely these will remain minor concerns/data presentation modifications.

Part III – Minor Issues: Editorial and Data Presentation Modifications

Reviewer #1:

This manuscript contains several technical errors. Therefore, it will be published once the following errors are corrected.
1. 1. Figure 1 and Supplementary Figure S1 display RVA levels in the small and large intestines for G5P [7] and G9P [13]. However, the p-values for these genotypes differ between the two figures. It is suggested that Figure 1 be deleted and replaced with Supplementary Figure S1.

AU: We appreciate the reviewer for pointing this out. The discrepancy in p-values between Figure 1 and Supplementary Figure S1 was due to an earlier version of the analysis being mistakenly used. The figures have now been corrected, and Figure 1 has been replaced with the revised version consistent with Supplementary Figure S1.

2. The first letter of words in a subtitle must be capitalized or lowercase.

AU: Revised as suggested

3. The authors used multiple GN piglets to examine RVA RNA levels in extraintestinal tissues. However, Figures 2D and 2F lack error bars, which suggests that the results are from only one pig. The authors should clarify this point. Is the statistical method used in Figure 2D the two-way ANOVA used in Figure 2B?

AU: We thank the reviewer for this comment. Figures 2D and 2F represent the frequency of RVA detection in extraintestinal tissues, not quantitative RNA levels, which is why error bars are not shown. The frequency of RVA detection across tissues from dpi 1–4 was assessed using Fisher’s exact test. We have now added this information to the figure captions for clarity.

4. Figure 4A: The positive fluorescence signals in each panel are hard to see. Organ or tissue names should be labeled. Additionally, each FITC-labeled lectin or HBGAs should be identified.

AU: Revised as suggested.

5. Figure 4B: Write the full names of each lectin and HBGA in the legend for Figure 4B.

AU: Revised as suggested.

6. Figure 5: You used 3-6 GN piglets in each group. However, you did not specify whether the pigs within these groups showed similar lung lesions. Therefore, compile a table with lung lesion indices for these pigs and include a description of these results in the text. Also, add a scale bar to each figure.

AU: We appreciate the reviewer’s helpful suggestion. Information on the lung lesion indices for individual piglets corresponding to Figure 5 has been compiled and included in **Supplementary File 1**. Additionally, the images in Figure 5 have been updated to include scale bars as recommended.

7. In the H&E-stained tissue in Figure 6, hematoxylin staining appears weak. This is probably due to inadequate tissue fixation in neutral formalin or insufficient hematoxylin staining. If possible, replace the image with one that shows stronger hematoxylin staining.

AU: We appreciate the reviewer’s observation. The quality of the H&E-stained images has been improved in the revised version. The updated figure now shows stronger hematoxylin staining and enhanced contrast between tissue structures, providing clearer visualization of histological details.

8. Describe the virus strain used in Figure 6.

AU: We appreciate the reviewer’s comment. For the in situ hybridization (ISH) representative image shown in Figure 6, we used the RVA strain G5P[7]

9. Instead of describing the magnification of each panel in the legend of Figure 6, include a scale bar for each panel.

AU: Revised as suggested.

10. Figure 6C legend: Jejunum showing linear positive signals (brown) along the villi surface for Rotavirus A RNA in situ hybridization.

AU: Revised as suggested

11. The pig schematic for necropsy in the scheme of Figure 7 should be replaced with a piglet.

AU: Revised as suggested

12. The same full names (abbreviations) are used repeatedly in Materials and Methods.

AU: Revised as suggested

13. There are no page numbers or line numbers throughout this paper, which makes it difficult to identify what needs to be corrected clearly.

AU: Revised as suggested

14. Reference #24 should probably replace the one described below in the Materials and Methods section, "Immunofluorescence for sialylated and non-sialylated glycans" (Lin et al., 2017).

AU: We thank the reviewer for pointing it out. We have replaced Lin et al., 2017 with Reference #24 in the manuscript.

Reviewer #2:

1. Please assign page and line numbers.

AU: Revised as suggested

2. Throughout the manuscript, the terms GF and Gnotobiotic are used. Please unify the terminology (e.g., consistently using “Gnotobiotic pigs” or defining GF at first mention and using the abbreviation thereafter).

AU: We thank the reviewer for pointing this out. We have unified the terminology throughout the manuscript: the term ‘germ-free (GF) pigs’ is defined at first mention, and the abbreviation GF is used consistently thereafter.

3. Please enlarge the font size in the figures. Several axis labels, legends, and annotations are difficult to read. Improving legibility would greatly enhance data interpretation.

AU: Revised as suggested

4. Figure 1: Consider using log-scale on the y-axis in panels A, B, and C to better illustrate variation across time points.

AU: Revised as suggested

5. Did any animals exhibit respiratory signs or clinical manifestations related to lung involvement?

AU: No animals exhibited respiratory signs or clinical manifestations related to lung involvement during the study. All gnotobiotic pigs remained clinically healthy, with no evidence of respiratory distress.

6. Please specify the viral genome copy number per dose in the inoculum for each RVA strain.

AU: In our study, the inoculum contained 2.24 × 10⁷ genome copies per dose for the G5P[7] strain and 4.61 × 10⁷ genome copies per dose for the G9P[13] strain.

7. Consider citing: Hou et al., 2025. "Innate immune sensing of rotavirus by intestinal epithelial cells leads to diarrhea." Cell Host & Microbe 33, 408–419. This study demonstrates that RV-induced diarrhea may occur in the absence of viral replication, suggesting a possible role of innate immune activation by viral particles. It is intriguing whether pathological change in lungs were induced by viral protein or genome lacking infectivity.

AU: We thank the reviewer for this insightful suggestion. We now have updated the Discussion section:

Interestingly, while we observed pathological changes in the lungs, the level of viral replication in this tissue was very low. A recent study by Hou et al. (2025) demonstrated that rotavirus-induced diarrhea can occur in the absence of active viral replication, suggesting that innate immune activation by non-infectious viral particles may contribute to tissue pathology. We have cited this reference in the revised manuscript to highlight this possibility.

8. Figure 4: Please add labels showing tissues.

AU: We have expanded the font size in Figure 4 to improve visibility of the tissue labels.

9. Related to Figure 4: Please specify which glycan receptors are relevant for each of the RVA strains used in this study.

AU: In this study, we focused on the common glycan receptors for RVA attachment and binding. We have now included glycan preferences for all strains used: G1P[8], G4P[6], G5P[7], and G9P[13]. Specifically, in the context of glycan-specific preferences, these RVA strains have been shown to replicate more efficiently in HBGA-H–expressing tissues compared to HBGA-A–expressing tissues.

Reviewer #3:

1. In Fig 1C and S1C, the scale of the y-axis makes it difficult to have a sense for G9 virus titers. Are they five-fold lower than G9? 10-fold? 100-fold? A log2 scale or a linear scale that encompasses a broader range of labeled values (and does not cut off error bars) should be used.

AU: We thank the reviewer for pointing this out. We have revised it as suggested.

2. In Fig 2A-B, data are initially presented as shaded grids that depict a range of values. Then, a subset of the results is displayed in bar-graph form. The results chosen for bar graphs might be only the subsets that include data with statistically significant differences. In several cases these subsets include tissues samples for which no viral RNA was detectable. It is also mentioned that lack of statistical significance might derive from high standard deviation, which is reasonable among animals. To increase transparency and share the wealth of information generated by infecting the animals, the authors should display individual data points, overlaid on the bar graphs, for all four time points for G5P[7] and G9P[13]. The same type of data display should be included in the supplement for the other two viruses in the study. Showing each virus on a separate graph might be helpful for appropriate y-axis scaling.

AU: Revised as suggested.

3. Please add labels for tissue and lectin types to Fig 4A. In Fig 4B, how many independent images were analyzed from each tissue for each lectin type? Did they come from one or multiple animals? Please display the individual data points overlaid on the bar graphs.

AU: We have now enlarged the fonts for the tissue, lectin, and antibody labels. For the corrected total cell fluorescence (CTCF) calculation, we used three animals for each staining, with one image analyzed per animal.

4. In Fig 5, which virus and time point are shown? At minimum, representative images of lungs from animals infected with the viruses most often discussed in the paper (G5 and G9) should be included. Were the features described observed in the lungs of all RVA-infected pigs or just some? If not all, what was the frequency for each virus?

AU: We thank the reviewer for the question. We have now included (Figure 5) representative images of lungs from animals infected with G5 and G9 viruses with multiple time points per virus shown. Notably, the gross lesions described in the paper were observed in all G5- and G9-infected animals, just with variation on the severity of the lesions between individual pigs.

5. In Fig 6, please indicate the time point and virus shown in each panel. It would be helpful to see representative images from animals infected with G5 and with G9. Higher resolution/zoomed in images are needed to clearly show vacuolar degeneration of enterocytes in the ileum (Fig 6B compared with 6A). What is the rationale for showing H&E staining in the ileum but viral antigen in the jejunum? Were none of the ileum samples virus antigen positive?

AU: We appreciate the reviewer’s detailed suggestions and have revised Figure 6 accordingly. -	The figure has been updated to include the corresponding time points and virus group for each tissue section. These details are now clearly indicated in the figure panels and legend. The ileum image (B) has been replaced with a higher-resolution image, and an inset has been added to highlight vacuolar degeneration of the enterocytes at the villus tips, and the figure C now shows the RNAscope in situ hybridization positive signs in an ileum section.

6. How were numbers of animals to be infected for the experiment chosen?

AU: The number of animals to be infected in this experiment was determined based on previous studies assessing rotavirus replication and pathogenesis in gnotobiotic pigs, balancing statistical power with ethical considerations. We selected a sample size sufficient to detect biologically meaningful differences in viral replication and tissue tropism while minimizing animal use in accordance with institutional animal care guidelines.

7. How conserved is the VP6 sequence targeted by ISH probes among the four tested viruses?

AU: The VP6 gene encodes a highly conserved protein among RVA strains and is species-specific, enabling clear differentiation between RVA, RVB, and RVC. The ISH probe used in this study targeted VP6 mRNA regions that are well conserved within each RV species ([doi.org/10.1177/1040638718817502](https://doi.org/10.1177/1040638718817502)). Previous studies have validated that such VP6-targeting probes effectively detected actively replicating RVA and reliably distinguished it from other RV species.

8. Please provide additional details about the CCIF assay used to quantify RVA fecal shedding, including the cell type used for the assay and the antisera used to detect the viruses.

AU: We have updated the Material and Methods section as follows:

Briefly, serial 10-fold dilutions of processed samples in MEM containing 1 µg/mL porcine trypsin (Sigma-Aldrich, USA) were added to confluent monolayers of MA-104 cells in 96-well plates, centrifuged at 1,200 × g for 30 min, and incubated at 37°C for 18 hours. After fixation of cells with 80% acetone, monoclonal RVA-VP6-specific antibodies (RV RG23B9C5H11) diluted 1:100 in PBS were added and incubated overnight at 4°C. Following PBS washes, secondary FITC-conjugated goat anti-mouse antibodies (SeraCare Life Sciences, USA) were applied.

9. Please describe how working concentrations/dilutions of lectins and antibodies used in the study were selected/optimized

AU: The working concentrations and dilutions of lectins used in this study were selected and optimized following the approach described by Rebelo et al.(Rebelo et al. 2021). Briefly, for lectins, we tested a range of concentrations in combination with haptenic sugars and selected the highest dilution that provided a clear signal with minimal background. For HBGA antibody staining, we included non-relevant antibodies of the same Ig class to confirm specificity of the staining.
